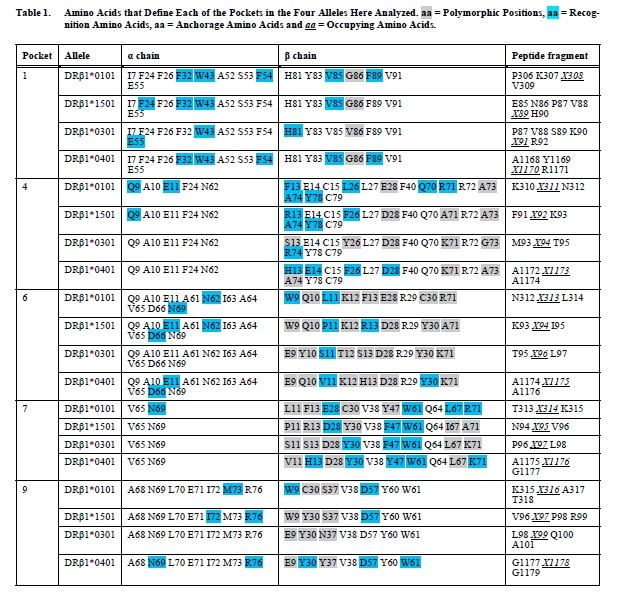# Quantum Chemical Analysis of MHC-Peptide Interactions for Vaccine Design

**DOI:** 10.2174/138955710791572488

**Published:** 2010-07

**Authors:** W.A Agudelo, M.E Patarroyo

**Affiliations:** 1 Fundación Instituto de Immunología de Colombia (FIDIC), Bogotá, Colombia; 2 Grupo de Química Teórica, Universidad Nacional de Colombia, Bogotá, Colombia; 3 School of Medicine and Health Sciences, Universidad del Rosario, Bogotá, Colombia; 4 Facultad de Medicina, Universidad Nacional de Colombia, Bogotá, Colombia

**Keywords:** Computational quantum chemistry, human leukocyte antigens, major histocompatibility complex, molecular electrostatic potentials, vaccines.

## Abstract

The development of an adequate immune response against pathogens is mediated by molecular interactions between different cell types. Among them, binding of antigenic peptides to the Major Histocompatibility Complex (MHC) molecule expressed on the membrane of antigen presenting cells (APCs), and their subsequent recognition by the T cell receptor have been demonstrated to be crucial for developing an adequate immune response. The present review compiles computational quantum chemistry studies about the electrostatic potential variations induced on the MHC binding region by peptide’s amino acids, carried out with the aim of describing MHC–peptide binding interactions. The global idea is that the electrostatic potential can be represented in terms of a series expansion (charge, dipole, quadrupole, hexadecapole, etc.) whose three first terms provide a good local approximation to the molecular electrostatic ‘landscape’ and to the variations induced on such landscape by targeted modifications on the residues of the antigenic peptide. Studies carried out in four MHC class II human allele molecules, which are the most representative alleles of their corresponding haplotypes, showed that each of these molecules have conserved as well as specific electrostatic characteristics, which can be correlated at a good extent with the peptide binding profiles reported experimentally for these molecules. The information provided by such characteristics would help increase our knowledge about antigen binding and presentation, and could ultimately contribute to developing a logical and rational methodology for designing chemically synthesized, multi-antigenic, subunit-based vaccines, through the application of quantum chemistry methods.

## INTRODUCTION

In general terms, presentation of antigenic peptides to the TCR, a key process for inducing an adequate immune response against any foreign antigen, is mainly driven by two types of molecules encoded by the MHC genes located on the short arm of chromosome 6: (a) Class I molecules (MHCI) expressed by all nucleated cells, and (b) Class II MHC molecules (MHCII) expressed only by antigen presenting cells (APCs), which include macrophages, dendritic cells, Langerhans’ cells, B lymphocytes, monocytes, etc.

**Fig. (1):**
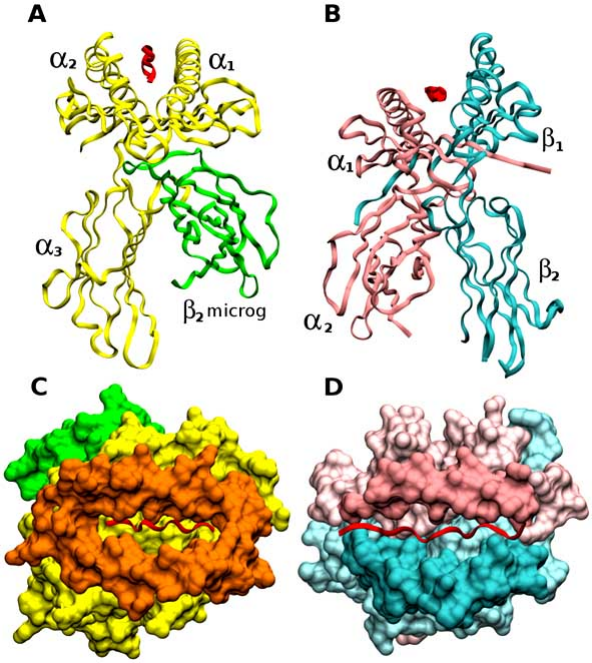
Structural and molecular characteristics of Major Histocompatibility Class I and Class II molecules. (**A**–**B**) frontal views; (**C**-**D**) top views, respectively. MHCI molecules (**A** and **C**) are formed by two molecular subunits: the α chain (shown in yellow), which contains the α_1_, α_2_ and α_3_ domains, and the β_2_-microglobulin (shown in green). In MHCI molecules, the binding groove (orange region in panel **C**) is formed exclusively by α chain residues. MHCII molecules (**B** and **D**) are formed by an invariant α chain containing α_1_–α_2_ domains (shown in pink) and a polymorphic β chain formed by β_1_–β_2_ domains (shown in cyan). In MHCII molecules, the PBR is formed by α and β chain residues. The antigenic peptide is represented as a red ribbon.

MHCI and MHCII molecules are structurally and functionally different. MHCI molecules are in charge of presenting antigens to T cell receptor to induce a cytotoxic immune response mediated by T lymphocytes and are formed by two molecular subunits: an α and a β chain (namely β_2_-microglobulin). Five domains can be distinguished within the α chain: α_1_, α_2_ and α_3_, a transmembrane (shown in yellow and orange in Fig. **1A** and **1C**), and a short intracellular tail, to which the β_2_-microglobulin (shown in green in Fig. **1A** and **1C**) is non-covalently bound.

Conversely, foreign antigens bound to MHCII molecules are presented to the T cell receptor and lead to antibody production. MHCII molecules are formed by two structural units: an invariant α chain containing α_1_–α_2_ domains (Fig. **1B** and **1D**, in pink) and a shorter polymorphic β chain formed by β_1_–β_2_ domains (Fig. **1B** and **1D**, in cyan). Both chains are anchored to the cell membrane by transmembrane domains and short intracellular tails.

**Fig. (2):**
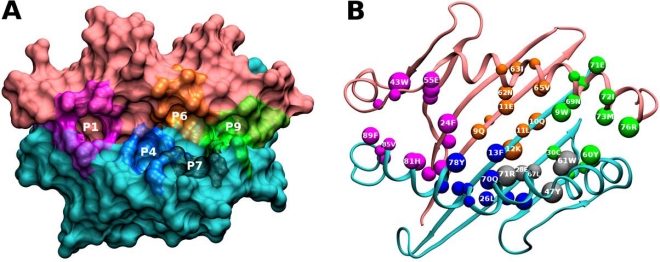
Top view of the binding groove of HLA Class II molecules (HLA-DRβ1*1501) formed by the α (pink) and β (cyan) chains. (**A**) Position of each Pocket (P1, P4, P6, P7 and P9) in the binding groove. (**B**) α- and β-chains amino acids that define each pocket, shown as balls according to the following color code: Pocket 1: fuchsia, Pocket 4: dark blue, Pocket 6: orange, Pocket 7: gray, and Pocket 9: green.

A binding groove or Peptide Binding Region (PBR) is formed at the extracellular end of both types of MHC molecules by two α-helices and a platform of β sheets inside which the peptide is anchored (Fig. **1A** and **1B**). Depending of the type of MHC molecule, the PBR is formed by different domains. In MHCI molecules, the PBR is formed by α_1_ and α_2_ domains (Fig. **1C** in orange) and is closed at both ends so that only peptides with a constant length of about 8–10 amino acids can fit inside such groove (Fig. **1C** in red). In MHCII molecules on the contrary, the PBR is defined by α_1_ (Fig. **1D** in pink) and β_1_ domains (Fig. **1D** and Fig. **2**, in pale blue), which form an open groove inside which peptides of variable length (between 12–20 amino acids) can be anchored (Fig. **1D** in red).

Five pockets can be distinguished in the PBR of MHCII molecules. As shown in Fig. (**2**), the peptide’s N-terminal residue is anchored inside a first ‘pocket’ located at the farthest portion of the PBR, denoted as Pocket 1, while residues towards the peptide’s C-terminus are anchored inside neighbor pockets named Pocket 4, Pocket 6, Pocket 7 and Pocket 9.

In humans, MHC molecules are referred to as Human Leukocyte Antigens (HLA). MHCI molecules are encoded by the HLA-A, B and C genes for which more than 450 variants have been reported. Similarly, three large groups of gene alleles or isotypes comprising more than 430 variants with similar molecular structures exist for human MHCII molecules, which are denoted as HLA-DP, DQ and DR, of which DR is the most polymorphic region [1].

The β chain of HLA-D Related molecules (HLA-DR) is encoded by the first gene of the DR subregion of the HLA class II locus, for which it has been named as HLA-DRβ1*. Sixteen alleles have been described for HLA-DRβ1* denoted as HLA-DRβ1*01–16, which comprise more than 300 variants and can contain between one (micro-polymorphism) to 30 amino acid sequence variations. The immune system’s ability to respond to the vast diversity of antigens it encounters during a lifetime depends on the different genetic variants harbored by an individual, which guarantees an appropriate and varied antigen binding/presentation [1].

The first stage, and probably the most important one in antigen presentation to induce antibody production, involves the recognition of the antigen by any of these HLA-DRβ1* molecules and the perfect fit of its anchoring residues inside the binding groove of the PBR, which is stabilized by a network of ~12 hydrogen bonds established between lateral chain residues of MHCII molecules and the antigen’s backbone residues. Depending on the time that the antigen remains anchored inside the groove of MHCII molecules, the TCR would form more stable and appropriate peptide–MHCII (pMHCII) trimolecular complexes and hence activate an effective immune response.

Since all these steps are fundamental for antigen recognition, and therefore for the activation of an adequate immune response, all these characteristics should be considered for developing a logical an rational vaccine development methodology, especially when dealing with multi-antigenic, minimal subunit-based, chemically synthesized vaccines.

It has been widely shown that peptide binding to the MHC groove is mediated by at least two key aspects: (a) recognition specificity, and (b) binding strength. Different experimental and computational models have been developed as an approximation to study these two aspects.

Experimental models make use of the data gathered by *in vitro* binding assays with purified HLA-DRβ1* molecules. When these molecules are exposed to a panel of peptides, only those peptides interacting specifically with purified molecules HLA-DRβ1* molecules are bound. The amino acid sequences of bound peptides can be determined by mass spectrometry (MS) once they are eluted from the purified MHCII molecules.

Two types of predictive tools can be constructed based on experimental data: (a) binding profiles and (b) mathematical/statistical models. Binding profiles describe which amino acids are most frequently found occupying a particular position in peptides binding to a specific MHCII allele and are used for building scoring systems (score matrices [2]). These score matrices are in turn used for designing linear prediction schemes based on the following hypotheses: first, that each position within the peptide contributes independently to the binding interaction; and second, that residues located on a given position within a peptide contribute equally to peptide binding, even if they belong to different peptides. These hypotheses are a good approximation to explore the peptide binding problem; however, they should be used with caution since it has been demonstrated that each position within a peptide has a different contribution to the peptide’s binding ability, as indicated by assays with truncated peptides, and glycine or alanine analogs [3]. In addition, an important limitation of this prediction scheme lies on the fact that if databases are redundant, the score matrix is biased and such bias can result in an over-fitting or under-fitting of binding values [4], both for false negatives (when no binding is predicted but the binding interaction has been evidenced *in vitro*) as well as for false positives (when binding is predicted but no evidence has been obtain experimentally).

To overcome the limitations shown by binding-motif-based methods, artificial intelligence models have been developed mainly based on non-linear mathematical/statistical models, of which artificial neural networks (NNs) are a classical example. In these models, the main construction hypothesis results from an initial alignment of peptide sequences used in the training of the neural network and sequences fed into the model. Due to the large number of parameters that have to be optimized, this type of models require of large input sets; an issue that especially in the case of MHCII molecules, is problematic since a sufficiently large binding dataset is not available.

Hidden Markov Models (HMMs) can be also used to describe nonlinear complex relationships among datasets. Although HMMs should be trained on and feed with large datasets, they have the advantage of not requiring a preliminary alignment of the input sequences.

All the aforementioned methods require of large databases and can operate at a maximum proficiency of 80% [4] (NNs and HMMs show the best performance). Nevertheless, it should be noted that these models have been designed almost entirely for MHCI molecules, and that they do not consider the structures of binding peptides and MHC molecules as a variable that can affect binding strength and specificity.

Structure-based models are constructed based on the information gathered from the crystal structures of MHC molecules loaded with a particular antigenic peptide, and do not need to be fed with large amounts of binding data. These models can be applied to general ligand–protein interactions and compromise less popular protein-structure modeling techniques that have higher computational costs. Several types of structure-based models have been applied to the MHC-peptide problem. One of these models, denoted as protein threading or side-chain conformational search, predicts unknown protein structures by using known protein structures. In the case of MHC–peptide complexes, the method compares the sequence of the query peptide to the one of the peptide co-crystallized with a particular Class I or Class II molecule [5]. Two aspects are of key importance for the structure-based approach: (a) the availability of an appropriate peptide structural template, and (b) the choice of a pair-wise potential table. The notion underlying this model is that the interaction energy can be expressed as the sum of energy-independent pair-wise interactions [6].

Computer-simulated ligand binding or docking is another structure-based model widely used in receptor–ligand studies. This model examines affinities by testing all possible binding complexes that result from modifying the ligand’s translational, rotational and conformational parameters in order to obtain the lowest energy binding complex. However, due to the combinatorial MHC–peptide problem implicit in this technique, numerous variations have been performed to docking techniques. Molecular dynamics and statistical mechanical simulations have been employed to model receptor–ligand interactions and predict the structure of a probable complex between the antigenic peptide and the MHC molecule [7].

One of the fields of structure-based models that has shown significant development in recent years is molecular recognition, an example of such which is the Quantitative Structure-Activity Relationship (QSAR) model developed by Doytchinova and Flower [8]. Our studies on the modification of the electrostatic landscape of MHC pockets are included within this field.

Our approach focuses on the theoretical study of the interactions between the regions of the HLA-DRβ1* PBR defined by experimentalists and structuralists as “pockets” and the peptide regions more closely interacting with each pocket. The model assumes that the interaction of the peptide’s fragments buried inside each pocket is independent from the interactions in the remaining pockets. For each HLA-DRβ1* pocket, we have been able to identify which peptide amino acids would have a significant interaction, obtaining a very good agreement with *in vitro* results.

## 1. ELECTROSTATIC LANDSCAPE AS A TOOL TO STUDY PROTEIN INTERACTIONS

One the most important problems of biophysical sciences is the study of macromolecular interactions between two proteins. When molecules are sufficiently close, they influence each other through forces of electrostatic nature formed by the distribution of positive and negative charges, named intermolecular forces. The rationale behind studying such interactions is to understand the dipoles localized on each of the molecules, since these dipole-dipole interactions are the main explanation for interactions between biomolecules. When there are no permanent dipoles, the attention is focused on the possibility of having induced dipoles or momentary dipoles caused by the movement of electrons, which would give rise to weak interactions, dispersion forces and repulsive interactions. However, dipoles are a fair but crude approximation to start with.

A spatial distribution of charges (protons and electrons) creates an electric scalar field called the Electrostatic Potential. At large distances, the Electrostatic Potential may be expressed as an expansion in powers of 1/r:

(1) $$V\left(r\right)=\frac{1}{{4\pi \varepsilon }_{0}}\left[q\left(r\right)+d\left(r\right)+Q\left(r\right)+\cdot \cdot \cdot \right]$$

where:

$q\left(r\right)=\frac{1}{r}\int \rho \left(r\right)d\tau$

dr=1r2∫ρrcosθ dτ

$Q\left(r\right)=\frac{1}{r^{3}}\int \rho \left(r\right)r^{2}\left(\frac{3}{2}\cos^{2}\theta -\frac{1}{2}\right)d\tau$

The first three terms in the series expansion of the electrostatic potential shown in Equation 1 correspond to the monopole or charge (q), dipole (d) and quadrupole (Q), respectively, and are given in polar coordinates (r and θ), where π is a constant (3.1416...) and ε_0_ is the vacuum permittivity constant. It can be observed that each of the multipoles depends on the distribution of the negative charge (electronic density, ρ(r)). Such distribution is calculated based on quantum mechanics.

Thus, the interaction between molecules is not only a matter of interactions between two dipoles, or a dipole and a charge, which would reduce the total interaction to just the first or two first terms in the former expansion, but it is instead an interaction between two complete fields of charge.

Our approach has consisted on using the whole electrostatic potential over both interacting molecules so as to understand interaction forces not as dipole–dipole interactions, but instead as V_A_(r) vs. V_B_(r) interactions, where V(r) is the whole electrostatic potential of a molecule. The electrostatic potential is a scalar field in the space associated with the molecule full of heights and valleys, designated as “*the electrostatic potential landscape*”. Electrostatic potentials may be calculated by computational chemistry methods at the same level of accuracy of the wave function for any particular level of theory.

Nevertheless, computing the energy of interaction between two charge distributions remains a difficult task and for molecules as large as MHC molecules and peptides, it is still a far more elaborated problem. We have focused on qualitatively describing the variations induced on the *electrostatic potential landscape* of each interacting molecule when the second molecule is in its vicinity. These qualitative variations allowed us to *classify* pockets and occupying peptides according to the main effects induced on the *electrostatic landscape*.

### 1.1. Understanding Changes in the Electrostatic Landscape: The Multipolar Approach

This model is based on charge variations determined by means of quantum mechanical calculations. By considering charges as punctual charges (q), the different multipolar moments (dipole d and quadrupole Q) can be calculated as follows: first, the value of Mulliken’s atomic partial charges [9] is calculated using the Hartree-Fock (HF) method implemented in Gaussian [10]. These calculations are performed for each pocket by systematically changing the occupying amino acid [11] by each of the 20 possibilities. Since each replacement is expected to induce variations on the pocket’s electrostatic landscape, it is reasonable to take into account the electrostatic changes produced in the peptide’s lateral chains.

In consequence, the charge of a residue can be estimated based on the net partial charges of each residue’s lateral chain atoms as follows:

qiaa=∑qside  chain  atoms

The magnitude of the dipole and quadrupole of each residue is also estimated based on the charges of the atoms that composed each lateral chain, considering the α carbon as the coordinate origin for each dipole vector and quadrupole tensor:

$\begin{array}{c} x^{'}=x-x_{C\alpha }\\ y^{'}=y-y_{C\alpha }\\ z^{'}=z-z_{C\alpha } \end{array}$

Calculating the dipole moment as:

(2) $$p^{\mathrm{aa}}=\sum_{k=1}^{N}q_{k}^{\mathrm{aa}}r_{k}$$

(3) $$d_{i}^{\mathrm{aa}}=\sqrt{p_{i}^{\mathrm{aa}}\cdot p_{i}^{\mathrm{aa}}}$$

and the quadrupole moment as:

(4) $$Q_{\mathrm{mn}}^{\mathrm{aa}}=\sum_{k=1}^{N}q_{k}\left({3x}_{m}x_{n}-r_{k}^{2}\delta_{\mathrm{mn}}\right)$$

$\begin{array}{c} x_{1}=x_{k};x_{2}=y_{k};x_{3}=z_{k};\\ r_{k}^{2}=x_{k}^{2}+y_{k}^{2}+z_{k}^{2} \end{array}$

(5) $$Q_{i}^{\mathrm{aa}}=\sqrt{\sum Q_{\mathrm{mn}}^{2}}$$

Given that the charge, dipole and quadrupole have different orders of magnitude, they are normalized before being compared according to the following expression:

(6) $$x_{i}^{\mathrm{norm}}=\frac{x_{i}-x_{\min}}{x_{\max}-x_{\min}}$$

Since the aim is to identity the main electrostatic potential differences that occur at the interaction between peptides and MHC molecules, we proposed the descriptors *S^aa^* and ${\mathrm{Dif}}_{\mathrm{Tot}}^{\mathrm{aa}}$ which are based on normalized multipolar moments:

(7) $${S_{i}^{\mathrm{aa}}=\left[\left(q_{i}^{o}+q^{e}\right)+\left(d_{i}^{o}+d^{e}\right)+\left(Q_{i}^{o}+Q^{e}\right)\right]}^{1/2}$$

(8) $${\mathrm{Dif}}_{\mathrm{Tot}}^{\mathrm{aa}}=\sum_{i}S_{i}^{\mathrm{aa}}$$

where $S_{i}^{\mathrm{aa}}$ is calculated for each pocket’s amino acid (*i*). This descriptor allows quantifying the electrostatic variations induced on the amino acids that define an occupied pocket (o) when the amino acid (*aa*) occupying such pocket is systematically replaced, taking the electrostatic landscape of the empty pocket (e) as a reference. The ${\mathrm{Dif}}_{\mathrm{Tot}}^{\mathrm{aa}}$ descriptor is calculated for each pocket according to the *aa* occupying it and allows sensing global variations on the pocket’s electrostatic behavior.

### 1.2. Graphs of Electrostatic Potential

This model has been applied to four MHC class II human molecules (HLA-DRβ1* alleles) for which a crystallographic model is available in the PDB protein data bank: HLA-DRβ1*0101 (PDB: 1dlh), HLA-DRβ1*0401 (PDB: 1j8h and 2seb), HLA-DRβ1*0301 (PDB: 1a6a) and HLA-DRβ1*1501 (PBD: 1bx2) [12-17].

One of the first and most important results is related to the global electrostatic modifications (${\mathrm{Dif}}_{\mathrm{Tot}}^{\mathrm{aa}}$) induced on each pocket. As can be observed in Fig. (**3A**) and Fig. (**3B**), there is a large average variation on the electrostatic landscape of the HLA-DRβ1*0101 molecule’s Pocket 1 (P1) when comparing the occupied pocket and the empty pocket, while such variation is lower and more diffused on the other four pockets (P4, P6, P7 and P9). It is expected for the electrostatic landscape to change abruptly when the occupying amino acid is charged, as shown in Fig. (**3A**) for Lys, His, Arg, Asp, Glu. Nevertheless, milder variations are also observed when the occupying amino acid is not charged, as indicated by the ${\mathrm{Dif}}_{\mathrm{Tot}}^{\mathrm{aa}}$ variance shown in Fig. (**3B**); which despite not being notoriously large, is not zero in any case. These small variations could explain the binding selectivity shown by certain amino acids.

**Fig. (4):**
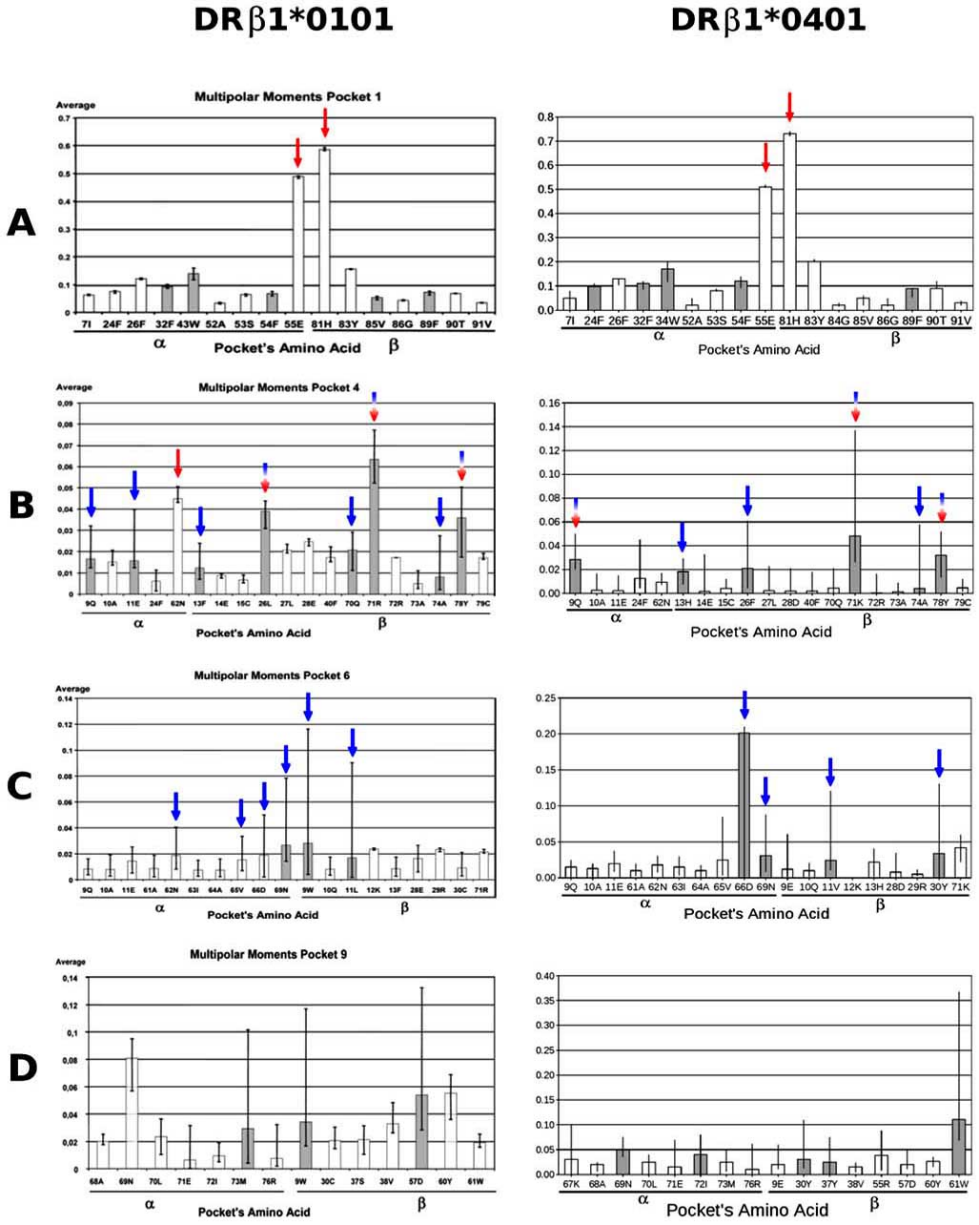
Electrostatic variations (*S^aa^*) on each of the amino acids that define a given pocket induced by each of the 20 possible occupying amino acids. The following cases are shown for Pockets 1, 4 and 6 defined in the HLA-DRβ1*0101-HA (left panel) and HLA-DRβ1*0401-Col II molecular complexes (right panels): (**A**) general or global variation (*anchorage*), (**B**) general variations (*anchorage*) mixed with specific variations (*recognition*) and (**C**) specific variations (*recognition*). See text for more detail.

An analysis of the electrostatic behavior on each of the amino acids that define a particular pocket when the pocket is occupied by each of the different amino acids is shown in Fig. (**4**). The graph shows the average *S^aa^* on each pocket’s amino acid with its corresponding variations indicated by length of the whisker. Three possible cases are identified in this graph: (a) a general variation of *S^aa^* induced by the presence of an occupying amino acid regardless of its identity (Pocket 1’s amino acids showing such behavior are indicated by red arrows in Fig. **4A**); (b) a large electrostatic variation exclusively associated to the identity of the occupying peptide (measured by length of variation bars and indicated by blue arrows in Fig. (**4C**) corresponding to Pocket 6); and (c) a mixed behavior showing a general electrostatic variation irrespectively of the presence of an amino acid inside the pocket, and notable variations related to the identity of the occupying amino acid (blue-red arrows in Fig. (**4B**) corresponding to Pocket 4).

These effects can be correlated with the binding capacity shown by the peptide’s residues buried inside the binding groove, which can be analyzed in two different ways: (a) nondifferential or nonspecific binding amino acids denoted as *anchorage* *amino acids* (on which when the electrostatic potential varies between an empty and an occupied pocket), and (b) differential or specific binding amino acids (on which the electrostatic potential varies exclusively as a result of the identity of the amino acid occupying the pocket). There are also combined effects caused by entry of a peptide portion and the identity of each particular amino acid, and are designated as *anchorage-recognition* *amino acids* (Fig. **4B**). Therefore, this methodology allows identifying the role played by the residues that define a given pocket and to classify such residues according to the electrostatic potential changes induced on them. A summary of each of the studied alleles is shown in Table **1**, where *anchoring amino acids* are shown in red, *recognition amino acids* in blue and *anchorage-recognition amino acids* in red-blue.

The performance of the model was evaluated by contrasting our findings with experimental data. For such purpose, we chose as the prototype occupying amino acid the peptide’s amino acid that was identified as being buried inside a particular pocket when the antigenic peptide was co-crystallized an MHCII allele. Such ‘best-fit case’ obtained for a particular pocket of a given HLA-DRβ1* allele by X-ray crystallography was denoted as the *ideal amino acid*. Electrostatic variations induced by the *ideal amino acid* and by other occupying amino acids were compared under the hypothesis that those occupying amino acids that induce electrostatic landscape variations comparable to the ones induced by the ideal amino acid should have similar binding characteristics.

**Fig. (5):**
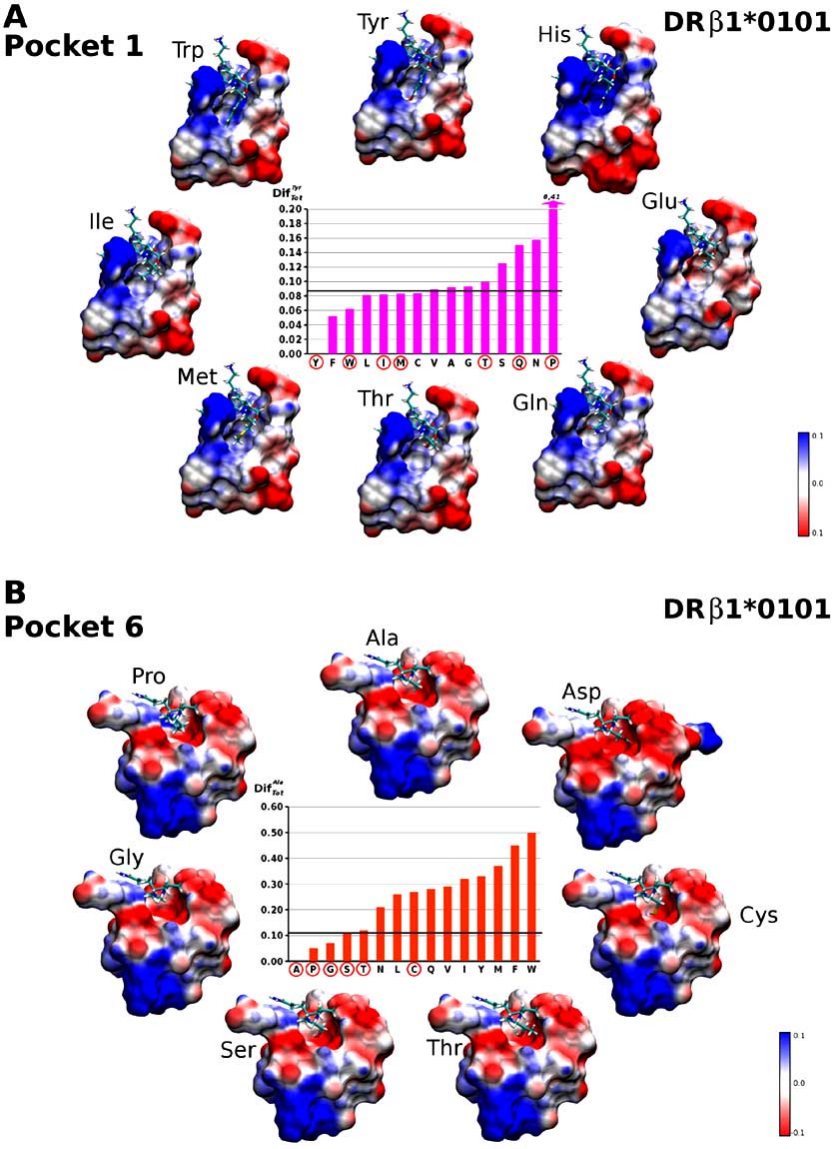
(*Inner plot*) Comparison of molecular electrostatic potential (MEP) changes expressed in terms of ${\mathrm{Dif}}_{\mathrm{Tot}}^{\mathrm{aa}}$ with respect to the ideal amino acids: tyrosine for Pocket 1 (**A**), and alanine for Pocket 6 (**B**). (*Around*) Electrostatic potential on the Michel Sanner’s Molecular Surfaces (MSMS [19, 20]) of Pockets, obtained by changing the identity of the occupying amino acid (encircled in red on the inner plot). Regions of negative electrostatic potential are shown in red, near zero in white, and positive in blue. Note the different degrees of MEP variation induced by some peptide amino acids and how such variation is quantified by the ${\mathrm{Dif}}_{\mathrm{Tot}}^{\mathrm{aa}}$ descriptor (*y*-axis in the bar plots).

In the case of the HLA-DRβ1*0101-HA’s Pocket 1 (Fig. **5A**), which is the largest and most important binding pocket in HLA-DRβ1* alleles, we found that the ideal occupying amino acid was Tyr (for more clarity, peptide amino acids are written in three-letter code and MHCII residues in one-letter hereafter). Other important occupying amino acids are Phe and Trp (all of which correspond to aromatic amino acids). Bearing in mind that the β-chain G86V dimorphism is located on Pocket 1 and that the allele herein analyzed harbors the 86G variant, its reasonable to hypothesize that other amino acids having smaller masses like leucine, isoleucine, methionine and cysteine can also fit inside this pocket regardless of residue being present in position β86 (valine or glycine).

By the same token, Pocket 6 (Fig. **5B**) is the most relevant pocket in HLA-DRβ1*0101 molecules since it is the smallest one. Our data for this pocket was contrasted to experimental data reporting Ala as the ideal occupying amino acid. Other ideal amino acids that have been reported include Gly, Ser, Thr and Pro. In the present work, the amino acids presenting a behavior more similar to the one shown by Ala were Pro, Gly, Thr and Ser (Fig. **7**); totally agreeing with experimentally obtained results [18].

It was difficult to optimize the side-chain geometry of the occupying amino acid for Pocket 6, mainly because there are two conserved amino acids in all Class II molecules within this pocket (α11E and α66D), which are very close to each other and whose interactions are stabilized by a network of hydrogen bonds. This situation prevents the interaction of this pocket with charged residues or with residues having long side chains, as it has been also found experimentally for most Class II alleles.

**Fig. (6):**
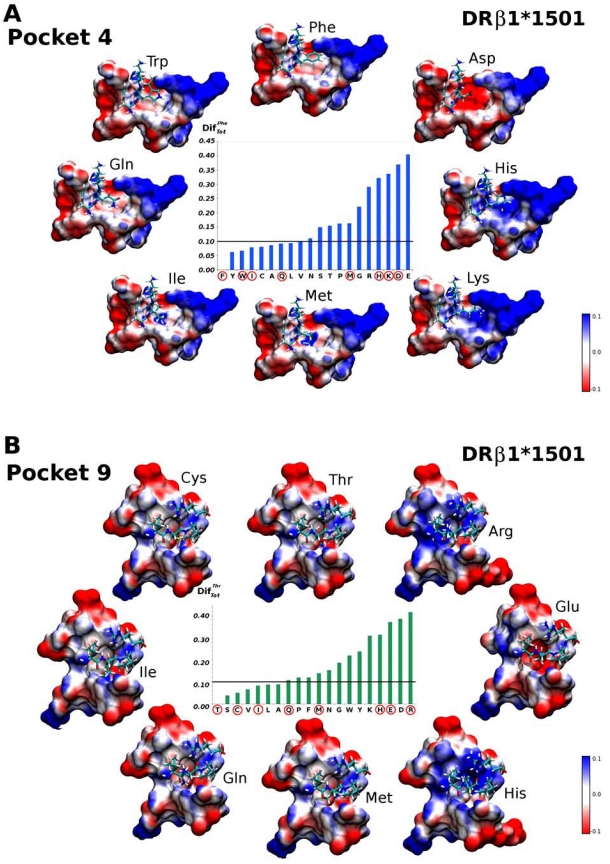
(*Inner plot*) Comparison of MEP changes expressed in terms of ${\mathrm{Dif}}_{\mathrm{Tot}}^{\mathrm{aa}}$ with respect to the ideal amino acid: Phenylalanine for Pocket 4 (**A**), and threonine for Pocket 9 (**B**) (see details in Fig. **5**’s caption).

**Fig. (7):**
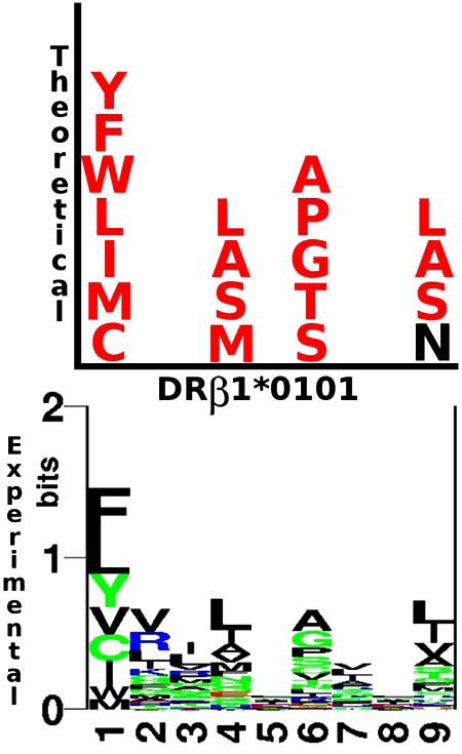
Comparison between theoretical data estimated based on the electrostatic landscape approach and experimental data reported as a logo plot [18] for the HLA-DRβ1*0101 allele. In the theoretical profile (*upper plot*), amino acid agreements are shown in red, while non-agreements are shown in black.

As another example of critical binding pockets, we can mention HLA-DRβ1*1501 (Fig. **6**). For Pocket 4, we found that Phe, Tyr, Trp, Ile, Cys, Ala, Gln, Leu and Val induce electrostatic variations similar to the ones induced by the ideal occupying amino acid Phe, which is in complete agreement with almost all the amino acids that have been reported to be binding motifs for this pocket [18], and to have a large affinity for this allele’s pocket (Phe, Tyr, Leu, Ile, Val and Ala). Large polar residues such as Lys or His, or short as Asp, introduce dramatic changes in the electrostatic landscape, as seen in Fig. (**6A**). Therefore, these amino acids are not likely to fit inside this pocket.

In Pocket 9, there is a genetic polymorphism where those alleles carrying the β57D variant, which establishes a salt bridge with α76R, have been found to bind apolar residues such as Leu, Ile, Val, Ala, Ser, Thr and Cys in experimental assays; this being in complete agreement with our results in the underlined amino acids. Those alleles carrying the β57 variant (replaced by A, S or V) bind charged residues like Glu, Asp and Gln in HLA-DRβ1*0405, or Arg and Lys in HLA-DRβ5*0101.

The mild and large electrostatic changes produced on HLA-DRβ1*0101 and HLA-DRβ1*1501 pockets can be observed in the MSMS surfaces [19] shown in Figs. (**5** and **6**), respectively. A visual inspection based solely on these maps is diffuse, whereas the descriptors herein proposed allow ‘quantifying’ changes on the electrostatic landscape and therefore to make a more precise analysis.

Fig. (**7**) shows the high correlation existing between occupying amino acids being identified experimentally for each of the HLA-DRβ1*0101 molecule’s pockets and the ones identified by our model, which leads us to conclude that the hypothesis herein postulated regarding electrostatic landscape variations allows explaining and helps understanding the binding properties of the peptide’s residues buried inside the groove of MHCII molecules.

## 2. GLOBAL ANALYSIS

When the results obtained for the four alleles discussed in this review are examined, common and particular features are observed among them. The average values of the ${\mathrm{Dif}}_{\mathrm{Tot}}^{\mathrm{aa}}$ descriptor for each studied allele show that the largest electrostatic changes occur on Pocket 1. This could be correlated with the importance that this pocket has in the anchorage of the peptide’s N-terminus, a key factor in the binding of antigenic peptides to the PBR. This feature is largely conserved among all HLA-β1* alleles, as shown by the fact that only the βG86V dimorphism is found for this pocket, but data shown in Table **1** indicate that α55E and β81H are also important for peptide binding. An example of the ${\mathrm{Dif}}_{\mathrm{Tot}}^{\mathrm{aa}}$ electrostatic variation on Pocket 1 is shown in Fig. (**4A**), where it can be observed that the occupying amino acid affects each of the various amino acids that define this pocket in different ways and magnitude. Predominantly, α55E and β81H are the most largely affected amino acids (Fig. **4A**), and this pattern is conserved among all Class II molecules studied to date [21, 22]. In particular and in support of our data, it is known that this latter amino acid is involved in the formation of a hydrogen bond with the peptide’s backbone.

The ${\mathrm{Dif}}_{\mathrm{Tot}}^{\mathrm{aa}}$ variation in the other pockets changes depending on the allele and peptide being analyzed. As observed in Table **1**, other amino acids located in different pockets are also relevant for the peptide’s anchorage, such as β74R in DRβ1*0301, α62N in DRβ1*0101, α71E in DRβ1*1501 / DRβ1*0401, and finally β71 in Pocket 6.

On the other hand, recognition amino acids (i.e. those that are sensitive to the identity of the occupying amino acid) and amino acids corresponding to polymorphic positions can be also distinguished in Table **1** (highlighted in blue and gray, respectively). Initially, it would be expected for polymorphic positions to be the only ones responsible for the pocket’s specificity for a particular occupying amino acid, but the analysis indicates that some conserved positions play also an important role in sensing variations in the identity of the occupying amino acid. The most notable case is shown in Pocket 1, where amino acids corresponding to conserved positions such as α32F, α43W and β85V are important for peptide recognition. Other important peptide recognition amino acids include the Pocket 4’s β13F, β26L and β74 polymorphic positions, Pocket 6’s β11 and β71 variants, and the Pocket 9’s β57D semi-conserved position.

## 3. CONCLUDING REMARKS

The results described in this mini-review highlight the plausibility of using *ab initio* methods to study biomolecular systems. Two methods have been commonly used for estimating molecular electrostatic properties: methods based on the Poisson-Boltzmann equation (PBE) and methods based on the electron density. In the first group of methods, it is necessary to assign a value to the atomic charges *a priori* (Field Force), which would remain constant with respect to different configurations that the molecule can adopt (a hypothesis that is not necessarily true). On the contrary, a major advantage of the second group of methods is that the value of the partial atomic charges is deduced by partitioning the wave function or density function (electronic population), and therefore the charges vary as the molecular configuration varies. Accordingly, *ab initio* methods take into account the electronic distribution and its variability; a highly desirable characteristic in the study of molecular interactions between biomolecules given that, as it is well-known, inter-and intra electrostatic forces involved in such interactions are mainly weak forces that result from the dispersion of the electric charge distribution [28,29].

The molecular descriptors proposed based on the multipolar expansion, which aims to describe the electrostatic landscape outlined by the molecular interaction, are of great value for evaluating the postulated hypothesis of similarity (similar electrostatic landscapes are associated to similar molecular activities) and addressing the complex problem of receptor–ligand complexes. This approach has shown good correlation with the experimental results of peptide binding to MHCII molecules [18], and has allowed identifying the key residues involved in this molecular interactions, thus showing great utility for understanding and elucidating the mechanisms involved in the process of receptor-ligand interaction as well as and for designing subsequent mutagenesis, bio-informatics and immunogenicity analysis of the key amino acid residues (both for the ligand and the receptor) among different MHCII alleles.

In the light of the promising results obtained so far for the peptide–MHCII binding problem, it would worth to keep on working with this approach and widen the electrostatic landscape model. For instance, much of the effort invested in calculating these type of biomolecular systems *ab initio* is not productive because the multipolar expansion series is restricted to the first three terms and therefore terms that would have to do mainly with widely distributed electric charge densities (non-point charges) are left out; moreover, there is no way of knowing how much information neglected. It would be more appropriate to work with the full scalar field defined by the electrostatic potential function, but that would require the use of mathematical functions with special properties (e.g. metric functions) to define molecular descriptors that could measure differences between such mathematical “objects” [30]. Moreover, it would be desirable to increase the level of *ab initio* single-point calculations, and to complementarily include Docking and Molecular Dynamics approaches to determine the configuration of the peptide amino acids that are anchored in MHCII pockets. Our current efforts focus on this purpose.

Research conducted in the search of a rational and logical vaccine development methodology has led us to identify some emerging principles for designing minimal subunit-based chemically synthesized vaccines [23]. One of these principles consists in modifying the critical host-cell binding residues of a candidate vaccine peptide specifically so as to improve its presentation to the TCR, bearing in mind that changes performed on the amino acid sequence result in structural and functional modifications due to the intrinsic structural complexity and degrees of freedom that relatively short peptides have (15–20 residues) [24-26]. This complex task would be greatly aided by theoretical models validated based on experimentally reported binding motifs and binding registers capable of describing the TCR–pMHCII molecular interaction system with good degree of accuracy. In this sense, studies such as the one reviewed in the present work are of key importance as they allow determining which amino acids can be replaced without altering the peptide’s overall binding characteristics but instead enhancing its binding properties.

Molecular recognition and molecular modeling are not only promising and developing areas of biochemical sciences but also of theoretical and computational chemistry. We are certain that the integration of these types of models, complemented with the results of HLA-DRβ1* molecules binding, T-cell proliferation and immunization assays, which we are currently performing in *Aotus* monkeys [23], will allow us to move forward in our endeavor to define theoretical principles for synthetic vaccine development.

## Figures and Tables

**Fig. (3):**
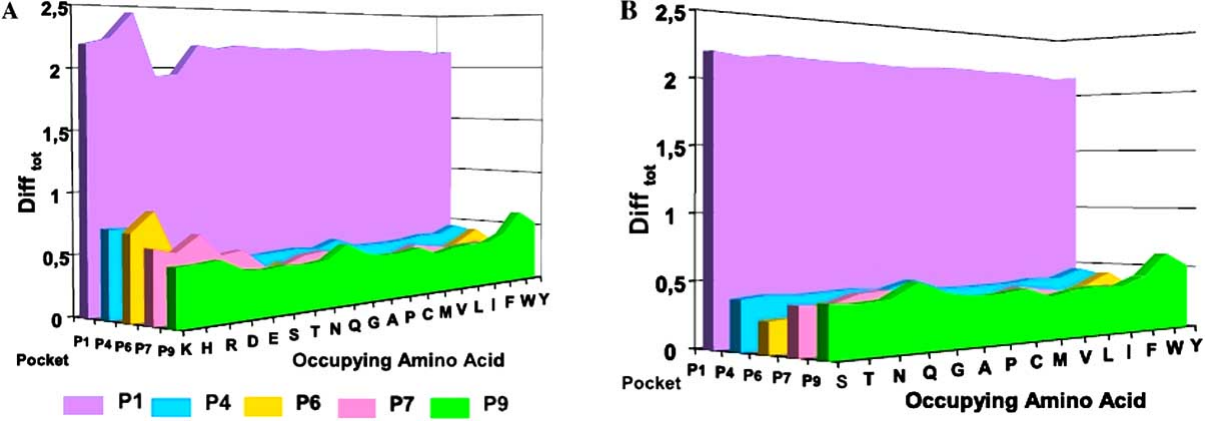
(**A**) Global electrostatic variations (${\mathrm{Dif}}_{\mathrm{Tot}}^{\mathrm{aa}}$ ) on HLA-DRβ1*0101’s pockets when being occupied by each of the 20 possible amino acids. (**B**) Same global electrostatic variations shown in panel A but without including charged occupying amino acids (lysine, histidine, arginine, aspartic and glutamic acids) to highlight variations due to neutral residues.

**Table 1:**